# Biofortification of baby corn: Integrating agronomic and genetic approaches to address zinc micronutrient deficiencies - global perspectives and future challenges

**DOI:** 10.3389/fpls.2025.1721421

**Published:** 2026-02-16

**Authors:** Saikrishna Mallipeddi, Venkateswarlu B, Sree Rekha M

**Affiliations:** Department of Agronomy, Agricultural College, Acharya N G Ranga Agricultural University, Guntur, Andhra Pradesh, India

**Keywords:** micronutrient deficiencies, zinc, baby corn, biofortification, agronomic & genetic biofortification

## Abstract

The central dilemma of the 21st century lies in sustaining nutrient-rich production under the pressures of rapid urbanization, weather abnormalities, and intensified agronomic practices. These factors have collectively heightened the burden on the remaining cropland to produce nutritionally dense food per unit area, while using the same amount of inputs. Moreover, modern high-yielding varieties often exhibit nutrient dilution, wherein increased productivity is accompanied by reduced micronutrient concentrations, thereby exacerbating global zinc (Zn) deficiency that affects more than two billion people worldwide. Zn deficiency manifests critically in cereal-based diets, with maize serving as a diagnostic indicator crop, where acute deficiency presents as characteristic the white bud symptom. To address this malnutrition regular dietary intake of essential nutrients is required in their natural form. A compelling strategy involves consumption of nutrient rich crops generated through the deliberate process of biofortification. Baby corn (Zea mays L.) presents unique fortifying opportunity through its rapid 60-day maturation cycle and wider adaptability throughout the year, facilitating quick nutritional interventions to support food security. Biofortification encompasses dual approaches: agronomic methods involving targeted Zn application through seed, foliar, soil, or combined delivery systems, and genetic strategies utilizing conventional breeding, molecular methods, and transgenic technologies. This review synthesizes the knowledge on Zn biofortification strategies in baby corn, critical aspects addressed include enhancing bioavailability, consumer acceptance, and economic viability. Future perspectives encompass the integration of agronomic and genetic approaches, emerging genomic tools, policy frameworks, and scalable implementation strategies essential for global biofortification success.

## Introduction

**Figure 1:**
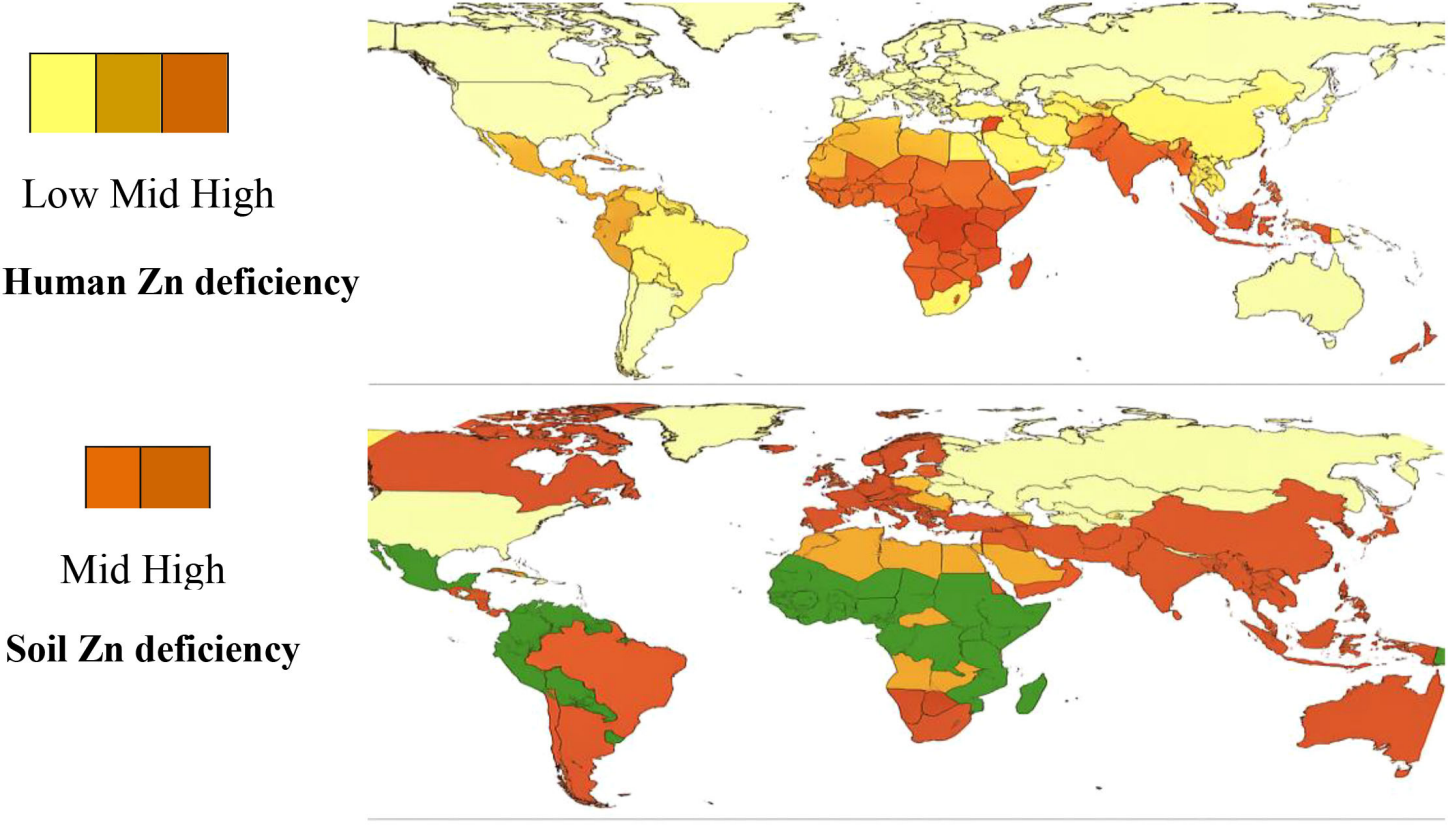
Human and soil Zn deficiency: geographical overlap (Adapted from Cakmak and Kutman, 2017).

Micronutrient malnutrition has become a critical concern in global food security discussions, as food security encompasses not only sufficient caloric intake but also adequate consumption of essential micronutrients (Gautam et al., 2021). Deficiencies in vitamin A, iron, and zinc are prevalent in developing regions, with South Asia and Africa accounting for 91% of the global micronutrient deficiency burden (**Figure 1**) . These regions with low soil Zn concentration shows a prevalence of Zn deficiency among humans suggesting a strong interrelationship among soil-plant and human health (Swamy et al., 2021). Zn deficiency alone affects up to two billion individuals worldwide and ranks fifth among health risk factors contributing to disease. It particularly affects children and expectant women, who are the strong future pillars of every nation (Baddam et al., 2025; Maret and Sandstead, 2006). The World Health Organization recognizes Zn deficiency as a significant contributor to global disease burden (Santos et al., 2017).

Zn, often referred to as the “metal of life,” is indispensable for numerous biochemical and physiological processes, including macromolecule metabolism. Its plays a critical role in diverse biological functions such as transcription, reproduction, immune defense, tissue repair, and sensory perception (McClung, 2019; Kaur et al., 2014; Roohani et al., 2013). The average dietary Zn requirement for adult men and women is estimated at 11 mg and 8 mg per day, respectively, however, actual intake often ranges between 6–7 mg/day (WHO, 2004; Brown et al., 2004). Zn deficiency induces several health problems in children, which may become chronic like stunted growth, weight loss, increased susceptibility to infections and mortality (Sagare et al., 2015). Zn deficiency syndrome in breastfed infants produced erosive skin changes like alopecia (Akhtar et al., 2018). Zn deficiency is also associated with delayed injury or wound healing (Kaur et al., 2014). Although Zn is essential, the human body cannot store it in significant amounts, necessitating regular dietary intake. This presents a challenge as cereals are inherently low in micronutrients, yet populations in developing countries remain highly dependent on them due to affordability and cultural habits. Consequently, Zn biofortification of cereals offers a sustainable solution (Alldrick, 2017).

Developing crops as indicator plants for Zn deficiency is essential for identifying areas with significant micronutrient limitations. Maize, often termed the “queen of cereals” due to its high productivity and nutrient potential, contributes approximately 39% of global grain production and it is also one of the leading edible crops in the regions having Zn malnutrition (Xu et al., 2021). Per capita consumption of maize is 290 g/day in women and 170 g/day in children of 4–6 years in the African countries, where maize is consumed as staple food crop (Andersson et al., 2017). In maize, Zn content varies significantly, averaging 20 μg/g in kernels with only 30% localized in the endosperm. While agronomic interventions like soil or foliar ZnSO_4_·7H_2_O application can increase grain Zn content to 35.6 mg/kg (Zhang, 2013), and the bioavailability is often constrained by anti-nutritional compounds such as phytic acid, fibre, phenolics and lignin. This underscores the necessity of simultaneously improving total Zn content and bioavailability (Nsabimana et al., 2024). Baby corn (also known as mini corn, young corn or candle corn) a young ear harvested before fertilization and considered as dual-purpose crop grown year-round in India, offering substantial domestic and export potential. While grain maize is a staple, baby corn baby corn offers a unique vehicle for nutritional intervention. Its short growth duration of approximately 60 days enables rapid crop turnover and timely agronomic manipulation, thereby presenting distinct opportunities for biofortification strategies aimed at enhancing micronutrient density (Singh et al., 2015).

Globally, micronutrient deficiencies are addressed through dietary diversification, supplementation, and industrial fortification. These are not necessarily mutually exclusive interventions, but these could be used as complementary approaches (Maqbool and Beshir, 2018). However, biofortification is recognized as a sustainable, acceptable, and cost-effective approach, integrating nutrition directly into staple crops to ensure continuous access to nutrient-rich foods, particularly in rural and low-income regions (Cheah et al., 2020; Stein (2010). Biofortification can be achieved agronomically, via seed priming, soil or foliar or their combination Zn applications during critical growth stages (Bhardwaj et al., 2022) and genetically through conventional breeding often enhanced by molecular tools or transgenic modification of genes encoding transporter and chelator (White and Broadley, 2011). It is an efficient and effective approach to get reliable results (Meenakshi et al., 2009). In increasing grain Zn concentration, wheat was the most responsive crop to leaf Zn spray (up to 83%), followed by rice (up to 27%) and maize (9%) (Cakmak and Kutman, 2018).

No single strategy can consistently achieve the desired outcomes due to the complex interaction of environmental and genetic constraints. Consequently, integrated or multi-dimensional breeding approaches are necessary to address these challenges effectively (Alloway, 2009). Evidence suggests that genetic improvement, when complemented by optimized Zn fertilization, can substantially alleviate micronutrient deficiencies, thereby improving dietary Zn intake and contributing to enhanced public health outcomes. Importantly, adequate Zn availability in soils is a prerequisite for accurately assessing genotypic responses through genetic approaches, highlighting the need to harmonize soil fertility management with breeding interventions (De Valença et al., 2017). Moreover, the cultivation of Zn-biofortified baby corn not only enhances human nutrition but also provides nutrient-dense fodder, which improves livestock health, productivity, and immune resilience. Zn concentrations in roots and leaves can reach 500–5000 mg/kg and 110–700 mg/kg dry matter, respectively, without compromising yield (Malesh et al., 2016). Emerging research also focuses on multi-nutrient biofortification (e.g., Zn with iron or provitamin A) and development of stress-tolerant, high-Zn cultivars. The cost-effectiveness of biofortification depends on adoption rates and consumption by target populations in forms that minimize nutrient losses (Vaiknoras et al., 2019).

At the field level, Zn accumulation can be enhanced either by increasing soil availability and uptake (agronomic strategy) or by improving internal translocation and sequestration (genetic strategy) (Das et al., 2019; Zaman et al., 2018). Although specific research on baby corn biofortification remains limited, insights from normal maize and other cereals provide a strong foundation for these comprehensive strategies. Given the critical importance of Zn for human health, this review focuses on developing Zn-biofortified baby corn genotypes through an integrated approach. Furthermore, it addresses the challenges associated with the dissemination of these Zn‐enriched genotypes to effectively combat malnutrition.

## Methodology

The literature reviewed in this article was identified through a structured narrative search approach designed to capture relevant research on zinc nutrition and biofortification in baby corn and maize. Searches were conducted using Scopus, Google Scholar, and ScienceDirect, covering publications from 2000 to 2025. Combinations of keywords related to crop type, nutrient focus, and experimental context were used, including maize, corn, baby corn, micronutrient, zinc, grain zinc, zinc concentration, nutrient composition, zinc sequestration, soil, fertilizer, organic manure, biofortification, nutrient enrichment, zinc accumulation, kernel genetics, digestibility, fodder quality, greenhouse studies, field experiments, and pot trials. Boolean operators (AND, OR) were applied to refine searches and ensure broad coverage of agronomic, physiological, and genetic aspects of Zn biofortification.

Titles and abstracts of the retrieved records were screened to remove duplicates, non-English publications, studies outside the defined time frame, non-food crop studies, and non-research items. Full-text articles were then examined for relevance to the scope of this review.

Studies were considered for inclusion if they:

i. Reported original experimental research on Zn biofortification in maize or baby corn under field, pot, or greenhouse conditions;
ii. Were published in peer-reviewed journals in English between 2000 and 2025;
iii. Included at least one control treatment without Zn application and one Zn-applied treatment; and
iv. Reported outcomes related to yield, grain Zn concentration, Zn uptake, or Zn accumulation/enrichment.

Studies were excluded if they were duplicate publications, short communications, book chapters, or conference abstracts lacking full experimental data, focused on crops other than edible, or addressed fortification approaches unrelated to agronomic Zn biofortification.

Following relevance-based screening and full-text assessment, approximately 80 articles were retained and synthesized in this narrative review.

## Agronomic biofortification

**Figure 2:**
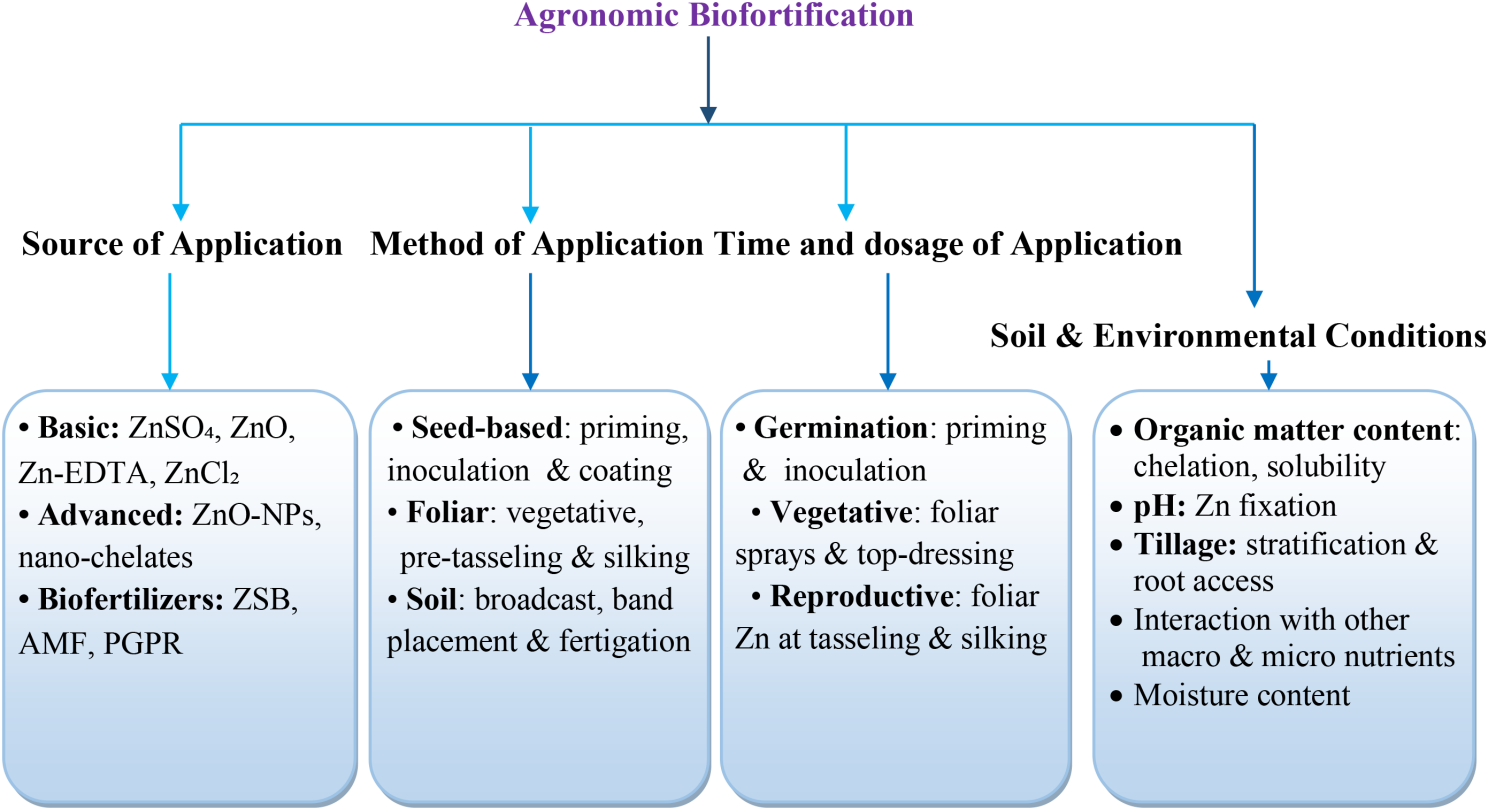
Schematic representation of key factors influencing agronomic biofortification.

Agronomic biofortification encompasses field-based interventions designed to enhance the concentration and bioavailability of essential micronutrients in edible plant tissues through targeted fertilizer inputs, soil amendments, and crop management practices. It represents the most immediate and scalable route to improving nutrient density in staple crops, particularly in regions, where micronutrient deficiencies coincide with highly weathered, low-fertility soils (Santos et al., 2024; Felix et al., 2023). Because nutrient acquisition is fundamentally governed by soil availability, root uptake kinetics, and subsequent xylem-phloem transport, the success of agronomic biofortification depends on synchronizing fertilizer source, method of application, and timing with soil chemical processes and plant physiological demand (**Figure 2**). Applied nutrients undergo complex bio-physiological transformations before partitioning into the developing tissues, processes that are strongly modulated by soil pH, organic matter, moisture regime, and texture (Hefferon, 2019). Understanding how these agronomic levers influence nutrient acquisition and sink loading is essential for developing sustainable, field-ready biofortification strategies (Oliveira et al., 2024).

However, crop responses to agronomic biofortification are highly variable due to strong genotype × environment × management (G×E×M) interactions (Santos et al., 2025; Bhardwaj et al., 2022). Root architecture, exudation profiles, transporter activity, phenological stage, and soil fertility status collectively determine, how efficiently nutrients reach edible tissues (Wasaya et al., 2018; Simić et al., 2020). Agronomic interventions therefore not only improve nutrient supply but also provide a standardized platform for identifying and screening genotypes with superior nutrient acquisition, internal transport, and cob loading efficiency (Cakmak and Kutman, 2017; Prom-U-Thai et al., 2020). When strategically aligned with breeding efforts, such interventions help disentangle genotypic differences under controlled nutrient conditions and enable more precise matching of fertilizer regimes to cultivar requirements (Da Silva et al., 2020). The factors affecting agronomic biofortification are subsequently discussed individually.

### Source of application

The choice of *Z*n source is a critical determinant of agronomic biofortification efficiency because the chemical form applied governs its dissolution, solubility, mobility in soil, root acquisition, and subsequent partitioning to edible tissues. The agronomic effectiveness of any Zn fertilizer is regulated by soil pH, carbonate content, organic matter, and moisture factors that control the transformation of Zn into soluble or fixed pools. Across multiple studies, including Carmona et al., 2020 and Bhatt et al., 2020, Zn fertilization aligned with soil chemical conditions has produced consistent increases in root and grain Zn concentrations, often exceeding 20% over untreated controls, underscoring that most sources can be effective when matched properly with soil constraints.

Among conventional sources, zinc sulfate heptahydrate (ZnSO_4_·7H_2_O) remains the global benchmark due to its high solubility (570–1000 g L^−1^ at 20°C) (Montalvo et al., 2016), rapid release of Zn^2+^ into solution (Jan et al., 2020), and comparatively low cost (Veena and Puthur, 2022). Its agronomic performance, however, depends strongly on soil pH. ZnSO_4_ dissolves readily in acidic to near-neutral soils (pH < 6.5), where Zn^2+^ remains mobile, but its effectiveness declines sharply in alkaline or calcareous environments (pH > 7.5) due to precipitation of Zn(OH)_2_; or ZnCO_3_and strong adsorption onto CaCO_3_surfaces (Fonseca et al., 2020). In these high-pH soils, chelated sources such as Zn-EDTA maintain Zn in soluble form through strong ligand stabilization (high stability constant), preventing fixation and remaining effective up to pH 9-10 (Javid et al., 2021). Despite their superior efficiency, chelates are three to four times more expensive than sulfate-based fertilizers, limiting their adoption in large-scale systems (Vattani et al., 2021). In contrast, ZnSO_4_ continues to be the most economical option in tropical and subtropical regions dominated by acidic or mildly acidic soils (Oliveira et al., 2024).

Other inorganic sources include zinc oxide (ZnO) and zinc chloride (ZnCl_2_;). ZnO is attractive for its low cost and slow-release characteristics but has very low solubility under neutral to alkaline conditions, restricting its effectiveness unless finely micronized or engineered into nano-scale formulations (Kachinski et al., 2022). *Zn* chloride, although highly soluble, is used sparingly due to risks of chloride toxicity, leaf scorching in foliar sprays, and soil salinity accumulation, and is therefore limited mainly to liquid fertilizers and fertigation systems (Ponce-Garcia et al., 2019).

Moreover, the penetration efficiency of applied Zn is governed by the contact surface area. Consequently, enhancing the retention and spreading of foliar droplets is critical. In recent years, nano-formulated Zn fertilizers, such as ZnO nanoparticles (ZnO-NPs) or chitosan-encapsulated complexes, have emerged as promising alternatives. Their exceptionally high surface-area-to-volume ratios enhance reactivity, foliar adherence, and penetration (Milani et al., 2015; Kumar et al., 2021). Studies in maize and wheat demonstrate that foliar-applied Zn nanoparticles can increase grain Zn concentrations by up to 36%, equivalent to large doses (≈400 mg kg^−1^) of conventional ZnSO_4_, while enabling gradual release and improved performance under heat or moisture stress (Jalal et al., 2023). Nonetheless, field-scale validation is still required to quantify fertilizer-saving potential, long-term effects, and bioavailability outcomes relative to standard ZnSO_4_·7H_2_O (Dapkekar et al., 2018). Moreover, the economic viability of nano-Zn remains to be validated for smallholder systems (Faizan et al., 2021).

Biological approaches further complement mineral fertilizers. Only 1-5% of applied Zn is typically taken up by plants, with the remainder immobilized in the soil matrix (Zhao et al., 2011). Zn-solubilizing bacteria (ZSB), including Bacillus, Pseudomonas, Rhizobium, and Azospirillum spp., enhance Zn availability by solubilizing ZnO, ZnCO_3_, Zn_3_(PO_4_)_2_;, and ZnS through secretion of organic acids, siderophores, and proton extrusion (Inacio et al., 2024). Studies by Jalal et al., 2022 and Santos et al., 2025 show that ZSB improve Zn partitioning indices and support Zn acquisition particularly in neutral to alkaline soils, where chemical fixation is high. Diazotrophic bacteria also contribute synergistically by mobilizing phosphorus, selenium, and other micronutrients (Poudel et al., 2023). However, microbial performance in tropical systems is constrained by high soil temperatures (40-50°C), rapid desiccation, and limited survival, although heat-tolerant genera such as Bacillus, Azotobacter, and Azospirillum offer better resilience, improvements in carrier formulations and moisture management remain critical for reliable field performance (Mehdaoui et al., 2024; Philipo et al., 2021).

### Method of application

The method of Zn application plays a pivotal role in determining the pathway through which nutrients enter the plant, the extent of their mobility in the rhizosphere or phyllosphere, and the efficiency with, which they are ultimately transported to edible tissues (Bhardwaj et al., 2022). Aligning the delivery route with crop phenology and soil-plant physiological demand greatly enhances nutrient accessibility and cob level enrichment (Tabo et al., 2005). In agronomic practice, Zn is applied through three major pathways-seed priming or microbial inoculation, foliar spraying, and soil application-each operating through distinct mechanisms and offering unique opportunities for improving nutrient delivery.

### Seed priming/inoculation/coating

Seed priming involves controlled hydration (hydropriming), exposure to nutrient-rich osmotic solutions (osmopriming), or inoculation with beneficial microbes to activate early metabolic processes without radicle protrusion (Maqbool and Beshir, 2018). Priming restores membrane integrity, accelerates enzyme activation, and stimulates early expression of Zn-dependent pathways, thereby strengthening root growth and enhancing early Zn uptake capacity (Nciizah et al., 2020). The resulting improvement in seedling vigour promotes more effective Zn translocation to developing tissues (Guevara et al., 2021). ZnSO_4_-based priming has increased maize grain yield by approximately 27% and improved biological yield across several studies (Harris et al., 2007; Mohsin et al., 2014). Osmopriming with Zn solutions has also enhanced kernel Zn concentration in crops such as maize and beetroot. Carmona et al., 2020 and Mohsin et al., 2014 further demonstrated that integrating Zn priming with reproductive-stage foliar sprays increases kernel Zn concentration by up to 40-44%, illustrating the clear synergy between early and late nutrient delivery.

Nevertheless, priming responses depend strongly on genotype, seed coat permeability, osmotic potential, and priming duration (Farooq et al., 2009). Hard-seeded or poorly stored seeds often show limited response. Importantly, seed priming cannot overcome Zn fixation in high-pH soils, where supplemental foliar or chelated Zn is required (Moreno-Lora et al., 2023). Overall, seed priming is a low-cost, farmer-friendly strategy that enhances early vigor and complements subsequent foliar or soil interventions (Goteti et al., 2013).

### Foliar application

Foliar Zn application, which involves applying dilute nutrient solutions directly to leaves, offers a rapid means of correcting deficiency and enhancing kernel enrichment, particularly when soil Zn availability is restricted by high pH, carbonate content, or antagonistic nutrient interactions (Hassan et al., 2021). Foliar-delivered Zn bypasses soil fixation and is absorbed through cuticular aqueous pores, polar pathways, stomatal flooding, and trichome bases, subsequently diffusing into the epidermis and mesophyll (Xia et al., 2019). Uptake efficiency is influenced by leaf surface properties, solution pH, formulation type, humidity, and the use of surfactants that increase droplet spread and cuticular permeability. Phloem mobility of Zn increases, when reproductive sink strength is high, making foliar application particularly effective from pre-tasseling to silking, when root uptake declines due to competition for assimilates (Kachinski et al., 2022). Mosavifeyzabadi et al., 2013 reported yield gains of 16.5-21.6%, when foliar Zn was applied during pre-seed formation, especially when combined with nitrogen fertilization.

Foliar Zn proves highly valuable in low-Zn, alkaline, or P-rich soils, and in systems, where root activity decreases during reproductive stages (Stewart et al., 2020). However, concentrations must be carefully calibrated, excessive doses, inappropriate pH, or omission of adjuvants can cause leaf scorching and reduce canopy function (Aref, 2010). While reproductive sprays are effective for grain maize, for baby corn, the application window is shortened. Foliar interventions must be targeted at the pre-tasselling (VT) to early silking (R1) stages to maximize loading into the immature cob before harvest (Wei et al., 2012). Numerous studies (Kanwal et al., 2010; Prom-U-Thai et al., 2020; Inacio et al., 2024) consistently show that foliar + basal soil Zn applications outperform either method used alone, confirming complementary uptake pathways.

### Soil application

Soil application provides a sustained supply of Zn^2+^ to the root zone across the growing season and remains the most widely practiced delivery method (Ali Raza et al., 2021). Once applied, Zn fertilizers undergo dissolution, sorption, precipitation, and diffusion or mass flow toward the root surface, processes governed by soil pH, CaCO_3_content, organic matter, moisture regime, texture, and background nutrient status (Fonseca et al., 2020). Zn remains relatively soluble in acidic soils (Obaid et al., 2022) but rapidly becomes fixed as Zn(OH)_2_; or ZnCO_3_in neutral to alkaline soils, particularly those rich in carbonates (Anwar et al., 2022). High phosphorus availability can further suppress Zn uptake through P-Zn antagonism, whereas soil organic matter and root exudates (citrate, malate) can enhance Zn desorption and mobility (Zhang et al., 2013).

Placement also greatly influences Zn efficiency. Broadcast applications in calcareous soils often lead to rapid fixation, whereas banding or localized subsoil placement concentrates Zn near the active root zone and minimizes losses (Cakmak, 2008). Several studies (Imran et al., 2016; Jerlin et al., 2017; Kihara et al., 2020) confirm that aligning placement, dosage, and timing substantially improves Zn uptake and cob Zn enrichment. Soil application therefore serves as the foundational platform for biofortification but often requires integration with foliar or seed-based approaches under high-pH or nutrient-antagonistic conditions (Sanchez-Rodríguez et al., 2021). **Table 1** summarizes how source, application method, dose, soil type, and timing influence yield and ear/grain zinc concentration.

**Table 1:** Summarizes how soil type, application method, form, dose, and timing influence yield and ear or grain zinc concentration.

Experimental setup	Treatment protocol				Increased (%) of		Key physiological remarks	Reference
	Form	Method	Dosage	Application stage	Yield	Ear/grain Zn		
Pot-calcareous loam	ZnSO_4_	SA	12 mg Zn kg^-1^ soil	B	45 FB, 70 DB	57 S	Hybrids>Inbreds	Ali Raza et al., 2021
Field-slightly alkaline	ZnSO_4_	SA FA	0, 12.5, 25 kg ha^−1^ & 0.2% ZnSO_4_	B & FA 15, 30 DAS	45 E	82 E	Soil + foliar > soil or foliar alone	Saikrishna et al., 2025
Field-sandy clay loam	ZnSO_4_	SA FA	0, 25 & 37.5 kg ha^-1^ & 0.5% and 1.0% ZnSO_4_	B & FA 20, 40 DAS	24 G 26 FB	–	Non-linear response: Zn >25 kg/ha Foliar-only < combined soil + foliar Soil Zn ↑, NPK unchanged	Amutham et al., 2023
Field-sandy loam	Nano ZnO (ZnONPs)	SP	20 & 40 mg L^-1^	B	39.8 G	259.8 S	Zn enrichment: nano-Zn > ZnSO_4_ *Zn* uptake: 20 > 40 mg/L ZnONPs	Tondey et al., 2021
Pot-calcareous	ZnSO_4_	SA	3 mg Zn kg^−1^ soil	B	54.7 G	58.8 G	Zn uptake: Joint P+Zn Alone: P ∝ Zn	Sanchez-Rodríguez et al., 2021.
Field-calcareous alluvial	ZnSO_4_	SA	2.3,5.7, 11.4, 22.7, 34.1 (kg Zn ha^-1^)	B	16.7 G	74.9 G	Needed high Zn 22.7 to 34.1 kg/ha to improve kernels efficiency. Selectioning of cultivar is crucial.	Liu et al., 2020
Field-red sandy loam	ZnSO_4_	SA, SP FA	10 kg Zn ha^-1^ 0.6-1.2% Zn 0.05% Zn	B	138 E	28.6 E	Soil + foliar > soil or foliar alone	Palai et al., 2018
Field-deep red	RS BS AB & P	SP	8 kg Zn ha^−1^ (residual) & 150 and 200 mL ha^−1^ (liquid inoculant)	B	17.1 E	16.7 G	Inoculation + residual ZN > inoculation or residual alone	Jalal et al., 2022
Pot-soil: sand (8:2)	ZnSO_4_ AM	SA	20,40,60,80, 100,120 Zn mg kg^−1^ & 10g inoculum/pot	B	42.2 G	56.5 G	Optimum dose: Zn20 mg kg^−1^ + AM; ≥80 mg) cause toxicity	Saboor et al., 2021
Field	ZnSO_4_	SA FA	7.5 kg Zn ha^−1^ & 0.75%)	B, FA at V, R	–	67.7 G	Soil-only application less efficient; foliar alone insufficient; combined strategy required	Jan et al., 2020

SA, soil application; FA, foliar spray; SP, seed priming/coating/treatment; PS, presowing; B, basal; RS, residual Zn; BS, bacillus subtilis; AB, Azospirillum Brasiliense; P, Pseudomonas; AM, arbuscular mycorrhizal; B, basal; V, vegetative; R, reproductive; FB, fresh biomass; DB, dry biomass; E, ear; G, grain; S, shoot; DAS, days after sowing.

To address the limitations of soil fixation, fertigation offers a precision agronomic alternative. By injecting water-soluble Zn sources (e.g., Zn-EDTA or solubilized ZnSO_4_) directly into the irrigation stream, growers can maintain a continuous available Zn within the active rhizosphere (Yan et al., 2021). This method significantly enhances availability through a slow, regular supply that matches the crop’s rapid vegetative uptake flux Lu et al., 2021).

Despite the proven efficiency of fertigation in horticultural crops, its application especially for Zn in baby corn/maize remains significantly underexplored (Du et al., 2024). However, practical implementation requires careful management of ionic antagonism. Simultaneous injection of Zn with phosphorus-rich fertilizers must be avoided to prevent the precipitation of insoluble Zn-phosphates, which can clog emitters and reduce bioavailability. Regarding economic feasibility for smallholder farms, the cost-effectiveness of water-soluble Zn sources, which are significantly costlier than granular sulfates remains a critical gap that requires further research.

### Time and dosage of application

Timing of Zn application is fundamental to maximizing uptake and ear enrichment because Zn demand in maize follows distinct physiological peaks. The early vegetative period (V1-V3) is characterized by intensive cell division, high activity of Zn-regulated membrane transporters (ZIP family), and chlorophyll synthesis, deficiencies during this window manifest as “white bud,” indicating impaired photosynthetic development and restricted meristem activity (Inacio et al., 2024; Prom-U-Thai et al., 2020). At this stage, spatial matching between root distribution and Zn availability is essential, as the young root system remains shallow and limited in exploratory capacity (Amutham et al., 2023). Localized placement, such as banding or subsoil ZnSO_4_ application, increases root-Zn contact, as demonstrated by Soleimani, 2012 and Zhang et al., 2013, who observed improved root proliferation up to 30 cm depth. However, in neutral to alkaline or P-enriched soils, Zn fixation constrains soil-based delivery, necessitating integration with foliar or chelated sources for effective biofortification (Mohsin et al., 2014).

In baby corn early vegetative period (V1-V3) sets the foundation for uptake, while the transition to reproductive stages (VT-R1) represents the final opportunity for enrichment (Tremea et al., 2024; Guevara et al., 2021). Unlike grain maize, where applications can continue through the grain-filling period (R2-R3), biofortification in baby corn must be front-loaded. Foliar applications are most effective, when timed specifically at tasseling (VT) and silking (R1) to coincide with the rapid sink expansion of the immature ear, just prior to harvest (Bouis, 2003; Welch and Graham, 2004; Huang et al., 2003).

In agronomic biofortification, dosage refers to the nutrient quantity applied per unit area that maximizes crop response without inducing toxicity or unnecessary expenditure. Dose-response studies indicate that Zn uptake does not increase linearly, once soil sorption sites are saturated or plant demand is met, additional Zn mainly augments the soil pool without increasing cob Zn concentration (Kachinski et al., 2022). Martinez-Cuesta et al., 2021 showed that when maize grain Zn exceeds ~18 ppm, further Zn fertilization yields negligible benefit. Excess Zn may inhibit root elongation, interfere with Fe, Mn, and Cu homeostasis, reduce microbial activity, and suppress mycorrhizal colonization factors critical for sustainable nutrient acquisition. Effective Zn management therefore requires aligning fertilizer source, placement, and dosage with soil pH, carbonate content, organic matter, and moisture regime, and integrating soil application with foliar or seed pathways, where fixation risks are high (Izydorczyk et al., 2021).

General recommendations in maize-based systems include supplying ZnSO_4_·7H_2_O at rates equivalent to 10–12 kg Zn ha^−1^ (≈50 kg ZnSO_4_·7H_2_O ha^−1^) in normal soils every two to three seasons, and increasing rates to 20–25 kg Zn ha^−1^ (≈100 kg ZnSO_4_·7H_2_O ha^−1^) for alkaline or calcareous soils to offset fixation losses (Costa et al., 2023). Foliar ZnSO_4_ solutions of 0.2–0.5% are commonly applied two or three times during critical growth stages, with higher concentrations avoided due to risks of leaf scorching and photosynthetic reduction (Anwar et al., 2022). However, the precise soil-foliar dose combinations required to consistently achieve nutritionally meaningful ear Zn concentrations remain insufficiently quantified and vary across genotype × environment × management conditions (Cakmak, 2008). Robust dose-response experimentation across diverse field environments remains essential for refining cost-effective and sustainable Zn biofortification strategies (Manzeke-Kangara et al., 2021; Zhao et al., 2014).

### Soil and environmental influence on agronomic biofortification

Soil and environmental conditions exert a decisive influence on the success of agronomic biofortification because they regulate Zn solubility, mobility, and root accessibility through their effects on soil chemistry, biological activity, and water dynamics. Key determinants include soil organic matter, pH, tillage-induced stratification, background nutrient status, and moisture regime. A systematic evaluation of these site-specific attributes is therefore essential for formulating location-responsive and sustainable Zn biofortification strategies in maize-based systems.

#### Organic matter

Soil organic matter (SOM) enhances Zn availability through multiple mechanisms: improving soil structure and moisture retention, increasing cation-exchange capacity, supplying chelating ligands that complex Zn^2+^, and stimulating microbial activity that transforms sparingly soluble forms into plant-available pools (Botoman et al., 2020). Field studies in India and Zimbabwe demonstrated substantial gains in grain Zn under FYM amendments, with increases nearly doubling relative to treatments lacking organic inputs (≈64% vs. 28%), and grain Fe concentrations also tending to improve (Goteti et al., 2013). However, in tropical regions, high temperatures accelerate organic matter decomposition and reduce the persistence of amendments, emphasizing the need for context-appropriate strategies such as residue retention, composting, mulching, and integrated soil fertility management (Imran et al., 2016). Further research is needed to distinguish the direct contributions of SOM to Zn solubility from its indirect effects mediated through microbial processes and water retention (Vanlauwe et al., 2011). Organic matter serves as a multifaceted regulator of Zn availability, but its effectiveness depends strongly on climatic conditions and residue management (Manzeke-Kangara et al., 2021).

#### Soil pH

**Figure 3:**
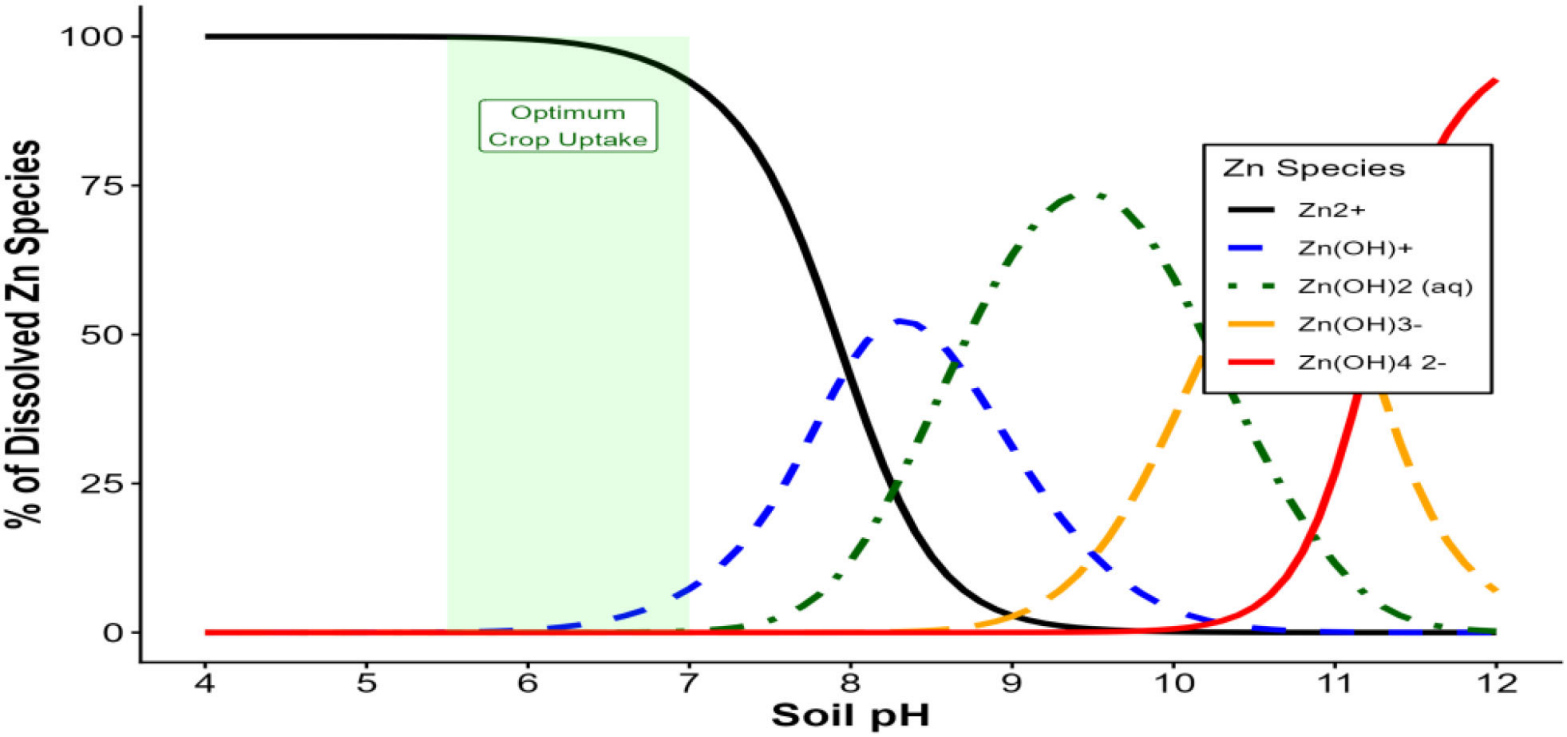
Theoretical equilibrium of Zinc species in soil solution plotted as a function of pH (Adapted from Guo et al., 2020).

Soil pH is among the strongest predictors of Zn availability because it governs Zn speciation, solubility, and adsorption to mineral surfaces. *Zn* is most available in mildly acidic to near-neutral soils (pH 5.5–6.5), where proton activity reduces Zn sorption to oxides and carbonates and promotes desorption into the soil solution (Rengel (2015). As pH increases, Zn becomes increasingly immobilized through precipitation as Zn(OH)_2_; and ZnCO_3_and through strong adsorption onto Fe/Al oxides and CaCO_3_surfaces (Guo et al., 2020). Conversely, extremely acidic soils may exhibit Zn toxicity or reduced availability due to intense weathering and depleted SOM. The figure serves as thereference (**Figure 3**). Managing pH through liming, gypsum, organic amendments, or placement strategies is central to optimizing Zn bioavailability across soil types (Almendros et al., 2013).

#### Tillage

Tillage influences biofortification primarily by modifying the physicochemical constraints of the rhizosphere rather than the plant’s uptake physiology itself (Wright et al., 2007). Studies show that when Zn fertilizer is applied, grain Zn concentration does not differ significantly among tillage systems, however, reduced or conservation tillage generally enhances Zn availability by maintaining organic matter, promoting surface stratification of nutrients, improving aggregation, and conserving moisture (Stanislawska-Glubiak and Korzeniowska, 2009). In contrast, intensive tillage accelerates organic matter decomposition, increases bulk density and pH, and disrupts rhizosphere stratification factors that reduce long-term Zn mobility and availability (Garcia-Marco et al., 2014). Conservation tillage provides indirect but meaningful benefits to Zn biofortification by improving soil structure, organic matter retention, and moisture dynamics.

#### Cross nutrient dynamics

Interactions between Zn and other nutrients strongly influence biofortification outcomes. High phosphorus availability can depress Zn uptake by forming insoluble Zn-phosphate complexes and by competing for uptake pathways (Sacristan et al., 2019), whereas adequate nitrogen supply enhances Zn acquisition by stimulating root proliferation and sink strength (Hui et al., 2019). Interactions with Ca^2+^, Mg^2+^, Fe^2+^/Fe^3+^, Mn^2+^, and Cu^2+^ influence Zn through competition for transporters, dilution effects, or synergistic mobilization depending on soil pH and carbonate content (**Table 2**). Importantly, the same nutrient combinations can produce contrasting outcomes under acidic versus alkaline conditions due to shifts in solubility, charge balance, and mineral precipitation (Goteti et al., 2013; Guo et al., 2020).

**Table 2:** Interaction of zinc with other nutrients influencing its uptake in both plants and soils.

S.no	Nutrient	Site of interaction & type	Mechanism	Critical ratio	Effect on accumulation	Reference
1.	Nitrogen	Root cytoplasm (Intracellular), Synergistic (+)	Enhanced synthesis of ZIP transporters and proteins that bind/transport Zn	–	Higher N availbility creates greater metabolic demand for Zn and it accelerates transport and accumulation	Amutham et al., 2023, Hui et al., 2019
2.	Phosphorus	Soil solution (Extracellular), Antagonist (-)	Precipitation of insoluble Zn_3_(PO_4_)_2_ in soil solution	P:Zn>400:1	Forms insoluble (Zn-P) minerals, preventing Zn from staying in soil solution for plant uptake	Zhang et al., 2021; Rietra et al., 2017,
3.	Potassium	Synergistic (Primary with in plant) (+) Antagonistic (at excessive K levels, root) (-)	Facilitates easy movement of Zn within the phloem results rapid accumulation in tissues	–	Improves long distance transport of Zn with in the plant	Pasley et al., 2019; Xue et al., 2014
			Excess K^+^ competes for root uptake sites and can dilute Zn concentration in plant tissues	–	High K supply promotes rapid biomass growth, leading to a ‘Dilution Effect’ of Zn concentration, rather than direct root inhibition.	Losak et al., 2011,
4.	Iron	Root, Antagonist (-)	Competitive inhibition for the IRT1 transporter, overlap of QTL regions suggests shared genetic uptake pathways	Fe: Zn>10:1	Competitive inhibition at root surface and compete for same transport proteins causing antagonism	Zhao et al., 2020
5.	Manganese	Root, Antagonist (-)	Due to size similarity both are try to enter the plant root by same transporter proteins leads to reduced uptake	Mn: Zn > 10	Entry points are filled with manganese and *Zn* can’t get absorbed by the plant	Li et al., 2019
6.	Copper	Root, Antagonist (-)	Cu binds more tightly to chelates and root transporters than Zn, physically displacing it	Cu: Zn > 0.5	High Affinity Displacement: Cu binding to root transporters with higher stability than Zn, physically blocking Zn entry.	Xie et al., 2019

#### Moisture content

Soil moisture is a major regulator of Zn availability because it influences Zn^2+^ solubility, the diffusion and mass-flow movement of Zn toward roots, and the continuity of root-soil contact. Adequate moisture enhances dissolution of applied fertilizers, especially highly soluble forms like ZnSO_4_·7H_2_O, and stimulates microbial activity and root exudation that mobilize Zn from mineral and organic surfaces (Oliveira et al., 2024). Evidence from wheat and maize shows that Zn fertilization combined with irrigation improves yield and water-use efficiency under moisture-limited environments, although little response may occur, where baseline moisture is already adequate (Kaya et al., 2023). Soil texture strongly modulates moisture effects: clay soils retain water and sustain Zn diffusion but may promote fixation depending on mineralogy (Bagci et al., 2007), whereas sandy soils lose moisture quickly, limiting Zn mobility (Alloway, 2009). In rainfed maize systems characterized by fluctuating moisture regimes, Zn availability is highly sensitive to soil water dynamics, making moisture management a critical component of biofortification strategies (Reed et al., 2012).

Taken together, agronomic biofortification offers a rapid, practical, and cost-effective pathway to enhance micronutrient density in baby corn, but its success depends on harmonizing fertilizer source, placement, and timing within the broader context of soil constraints, environmental variability, and genotype-specific nutrient-use efficiency. Integrating these agronomic interventions with complementary genetic strategies remains essential for achieving reliable and nutritionally meaningful improvements in edible tissues under diverse production conditions.

## Genetic biofortification

After improving the solubility and availability of Zn in the rhizosphere, the next critical step is to target the genetic control of Zn acquisition, transport, and deposition within the plant. Mineral nutrition in maize involves a series of closely regulated processes, governed by complex physiological and biochemical networks (White and Broadley, 2009). Genetic biofortification is a seed-based approach that uses plant breeding to increase the concentration and bioavailability of Zn in edible tissues, offering a cost-effective and sustainable strategy to alleviate Zn deficiency (Ibrahim et al., 2022). Once superior high-Zn genotypes are developed and deployed, they can provide recurring nutritional benefits over many years with minimal additional cost beyond seed purchase. This non-recurrent nature led the 2008 Copenhagen consensus to rank biofortification in 5^th^ position among the most cost-effective interventions for combating hidden hunger. (http://www.copenhagenconsensus.com/publication/second-copenhagen-consensus-biofortification-best-practice-meenakshi).

Maize is a particularly suitable target for Zn biofortification because it exhibits substantial inherent genetic variation for kernel Zn concentration, with values ranging from approximately 4 to 96 mg kg^−1^ in diverse germplasm, and an averaging approximately 20 mg kg^−1^ (Al-Khayri et al., 2016; Gupta et al., 2015). Based on estimated dietary Zn requirements and typical maize consumption patterns, HarvestPlus established a breeding target of about 33 mg kg^−1^ grain Zn (dry weight basis), implying an achievable gain of roughly 13 mg kg^−1^ over current baseline levels (Gashu et al., 2021). While baby corn has a lower absolute concentration on a fresh weight basis due to high moisture, the consumption of the whole ear (cob + immature glumes) and the lack of phytate may result in a comparable or superior net Zn absorption per serving compared to dry grain flour. Dissecting the genetic architecture underlying this variation is essential for improving breeding efficiency and facilitating the introgression of favourable alleles into elite Zn-enriched germplasm through conventional and molecular breeding methods (Goredema-Matongera et al., 2021; Gallego-Castillo et al., 2021). A comprehensive understanding of Zn homeostasis is therefore a prerequisite for effective genetic biofortification. Grain Zn concentration is a classical quantitative trait, controlled by many loci each contributing a small effect to the overall phenotype (Ndlovu et al., 2024). Quantitative trait locus (QTL) analyses across multiple populations and environments have identified on the order of 50–60 genomic regions associated with kernel Zn concentration, providing a foundation for mapping, selection, and deployment of Zn-enhancing alleles (Baxter et al., 2013). Continued QTL discovery, meta- QTL analysis, and fine mapping are needed to pinpoint consistent, stable loci suitable for marker-assisted selection (MAS), genomic selection, and eventual map-based cloning (Jin et al., 2013). In this regard, heritability estimates for kernel Zn in maize (typically 0.60-0.70) are encouraging. These moderate-to-high values indicate that a significant proportion of phenotypic variance is genetic, validating the prospects for rapid genetic gain despite the presence of genotype × environment interactions (Singh et al., 2021). In mature maize grain, Zn bioavailability is heavily constrained by phytic acid (phytate), an anti-nutrient that accumulates primarily during the mid-to-late grain filling stages. This Zn-phytate complexation significantly inhibits absorption in the human gut, making it a critical bottleneck for nutritional quality in cereals (Simic et al., 2012).

However, the physiology of baby corn presents a fundamentally different scenario regarding bioavailability. Harvested at the onset of silking-long before the activation of phytic acid biosynthetic pathways baby corn effectively bypasses this bioavailability barrier (Saikrishna et al., 2025). Since the physiological processes responsible for sequestering minerals into insoluble phytate complexes are not yet active, the Zn in baby corn remains in a highly soluble, bioavailable form compared to mature kernels. Despite this bioavailability advantage, actual enrichment remains a challenge. High availability is useless if the absolute concentration of Zn is low (Nelson and Meinhardt, 2011). Therefore, maximizing Zn density in baby corn relies entirely on the plant’s efficiency in source-to-sink translocation and the remobilization of nutrients from vegetative tissues to the developing ear. A thorough understanding of these uptake and transport mechanisms in normal maize is essential for baby corn biofortification. Consequently, the subsequent sections examine the genetic, molecular, and breeding frameworks that can be exploited to enhance both Zn accumulation and bioavailability in baby corn.

### Conventional hybridization for Zn biofortification

Conventional hybridization is a principal breeding strategy enhancing grain Zn concentration in cereals such as maize, wheat, and rice etc (Kaur et al., 2020). It relies on combining complementary genetic variation through controlled crossing, followed by selection for progeny that express superior agronomic performance and elevated micronutrient (Huang et al., 2006). The ultimate goal is to assemble favorable alleles controlling Zn uptake, translocation, remobilization, sequestration into a single cultivar. Because cob Zn is a quantitative trait under polygenic control, hybridization and subsequent line development are essential for assembling multiple small- to moderate-effect loci into adapted genetic backgrounds (Gaikwad et al., 2020). As emphasized by Welch and Graham (2004), defining precise breeding objectives is critical for success. For Zn biofortification, these objectives rest on three interdependent pillars (**Table 3**).

**Table 3:** Key breeding objectives for zinc biofortification and their evaluation criteria.

S.no	**Component**	**Key requirement**	**Evaluated by**
1	Stability	Consistent quality and quantity across diverse agro-climatic zones.	Multi-Location Trials (MLTs)
2	Bioavailability	Nutrient must be at levels absorbable by the human gut (not just present in the grain/ear)	*In-vitro*/*In-vivo* studies
3	Acceptability	Organoleptic traits (taste, aroma & texture) that drive grower and consumer adoption.	Sensory Panels & Participatory Varietal Selection (PVS)

Historically, the Green Revolution achieved monumental increases in crop productivity through improved agronomic practices and the selection of high-yielding semi-dwarf varieties. However, this singular focus on food security imperatives (yield per hectare) inadvertently deprioritized nutritional quality. A notable consequence of this selection pressure is the yield dilution effect, where modern cultivars exhibit lower micronutrient densities compared to landraces due to the physiological trade-off between carbohydrate accumulation and mineral sequestration (Marques et al., 2021). Consequently, maintaining yield while breaking this negative correlation remains a non-negotiable prerequisite for the public acceptance of biofortified varieties. To overcome these historical constraints, a superior genotype for Zn biofortification in normal grain maize must be designed according to a general high-Zn ideotype, characterized by (White and Broadley, 2011; Bouis and Welch, 2010):

•High Zn acquisition efficiency across diverse soil and environmental conditions, ensuring reliable uptake under both low- and high-availability scenarios.
•Enhanced xylem-phloem translocation capacity, enabling effective movement of absorbed Zn from roots to shoots and ultimately toward developing kernel.
•Efficient remobilization of stored Zn from vegetative tissues during kernel development, maximizing Zn loading even under late-season stress.
•Emphasis on nutrient bioavailability rather than kernel zinc concentration alone, recognizing that nutritional effectiveness depends on absorbable zinc forms.
•Strong consumer and farmer acceptance, including desirable kernel appearance, texture, processing and cooking quality, and agronomic traits needed for production and market uptake.

In the context of baby corn, however, this remobilization criterion requires distinct interpretation. It specifically refers to the rapid partitioning of nutrients to the ear during the vegetative-to-reproductive transition (R1), distinct from the senescence-driven remobilization typical of mature grain. Consequently, breeding targets for baby corn must prioritize early-stage uptake flux rather than the late-season stay-green traits. Nevertheless, understanding conventional kernel Zn accumulation patterns remains essential. Since baby corn is agronomically identical to standard maize until the point of harvest (immediately prior to fertilization), the fundamental metabolic pathways governing Zn uptake and initial translocation are conserved, providing the foundational baseline for enrichment strategies.

### Genetic variation and germplasm exploration

The fundamental prerequisite for successful biofortification lies in the magnitude of genetic variability available within the primary gene pool. This variability is the raw material that allows breeders to exploit transgressive segregation creating progeny that exceed parental nutrient levels (Ortiz-Monasterio et al., 2007). Consistent with large-scale surveys by the CGIAR, global maize germplasm exhibits a substantial range of kernel Zn concentration (approx. 4–96 mg kg^−1^), confirming that donor alleles are abundant in landraces and improved inbreds (Banziger and Long, 2000; Menkir, 2008). However, a critical bottleneck in exploiting this variation has been the high cost and low throughput of precision phenotyping (Long et al., 2004). Genetic variability for kernel Zn concentration in different studies are presented in **Table 4**.

**Table 4:** Genetic variability for kernel Zn concentration in different studies.

S.no	Place of evolution	Sorts of germplasm	No of germplasm	Kernel Zn concentration (mg/kg)	References
1.	China	Landraces, improved genotypes	7	5.4-18.9	Chao et al., 2023
2.	Nigeria	Inbred lines	109	11.6-95.6	Goredema-Matongera et al., 2021
3.	Croatia	Hybrid	28	16.0-23.6	Rasool et al., 2021
4.	Mexico	Inbred lines, Hybrid	251	16.8-39.5	Guo et al., 2020
5.	–	Core accessions	400	15.0-47.0	Maqbool et al., 2018
6.	Mexico	Inbreds	923	17.1-43.8	Thakur et al., 2015
7.	India	Inbreds	46	12.6-39.4	Pandey et al., 2015
8.	India	Inbreds	188	16.4-53.2	Mallikarjuna et al., 2014
9.	Nigeria	Inbreds	24	20.0-53.0	Guleria et al., 2013
10.	India	Improved genotypes	48	19.4-32.6	Guleria et al., 2013
11.	India	Inbreds	67	7.01-29.8	Agrawal et al., 2012
12.	Brazil	Inbreds	22	17.5-42.0	Queiroz et al., 2011
13.	Mexico		42	19.3-30.9	Pixley et al., 2011
14.	India	Inbreds	31	21.8-40.9	Chakraborti et al., 2011

The scalability of fortifying programs hinges on cost-efficient grain analysis, making high-throughput phenotyping for exploiting the vast genetic potential of maize germplasm. While inductively coupled plasma-optical emission spectrometry (ICP-OES) remains the gold standard for accuracy, the operational necessity for high throughput favours X-ray fluorescence (XRF) as a viable alternative (Pixley et al., 2011). Although XRF exhibits lower precision, its non-destructive nature and cost-effectiveness facilitate the mass screening required to capture genetic variability. Aligning with Goredema-Matongera et al., 2021, the optimal strategy is therefore situational, future pipelines should adopt a tiered framework, utilizing XRF to discard inferior progeny in early generations, while reserving ICP-OES validation strictly for advanced lines, where precise quantification is mandatory. This interpretation is further reinforced by Velu et al., 2014; Ihnat, 2003; Pfeiffer and McClafferty, 2007.

Once donor lines are identified, the next step involves examining the mode of gene action governing Zn accumulation. Understanding, whether these genes are additive (fixable) or non-additive (heterotic) dictates the breeding strategy. This assessment aligns with Long et al., 2004, as well as Pixley et al., 2011 and Chen et al., 2007, who observed significant general combining ability (GCA) effects, suggesting a preponderance of additive gene action. This consensus indicates that recurrent selection can effectively increase the frequency of favourable alleles in a population. Conversely, Qin et al., 2012 and Chakraborti et al., 2011 reported significant specific combining ability (SCA) and dominance effects in QTL mapping studies, implying that heterosis plays a crucial role in specific hybrid combinations.

This apparent contradiction is resolved by recognizing that gene action is often germplasm-dependent. As suggested by Menkir (2008), the most effective strategy involves a two-pronged approach, exploiting additive variance for population improvement, while capitalizing on non-additive effects (heterosis) during final hybrid development.

Despite the high genetic potential, the stability of Zn expression remains a challenge due to significant genotype × environment interactions. This phenomenon can be attributed to the sensitivity of Zn uptake to soil fertility, moisture regimes, and soil texture, which can obscure the true genetic value of a line (Katral et al., 2024). Nevertheless, reported heritability estimates remain moderate-to-high, indicating that genetic gains are feasible if supported by robust multi-environment testing (Luo et al., 2023; Agrawal et al., 2012). The Fe-Zn link a promising observation for future breeding is the strong positive correlation often found between Zn and iron (Zhang et al., 2017). Consistent with Gupta et al., 2015, this co-segregation suggests that breeders can simultaneously improve both minerals without needing separate breeding pipelines, improving the cost-efficiency of the program. To translate these genetic gains into farmer fields, the integration of international frameworks is indispensable. The collaborative backbone led by International centre for wheat and maize improvement (CIMMYT) and HarvestPlus has successfully bridged the gap between lab discovery and field deployment. CIMMYT has led targeted maize breeding for higher grain Zn (and provitamin A) for decades developing Zn-enriched germplasm, testing hybrids on-farm, and supporting regional deployment in Latin America and Africa. Consultative group on international agricultural research (CGIAR) centres and partners (CIMMYT, IITA, CIAT, IRRI, ICRISAT, and others) coordinate multi-centre research on germplasm, mapping, and capacity building, forming the multidisciplinary backbone for genetic and agronomic work (Johnson, 2018a; Andersson et al., 2017). National and regional institutes such as International institute of tropical agriculture (IITA) complement these efforts with agronomic trials and datasets that quantify fertilizer and management effects on grain Zn and Fe. Representative biofortified maize cultivars developed through these partnerships under conventional breeding are summarized in **Table 5**. Moving forward, national programs must leverage these pre-breeding resources to validate biofortified materials under local agronomic management, ensuring that the genetic potential for high Zn is not lost to poor soil management (Malesh et al., 2016).

**Table 5:** List of biofortified maize cultivars in the world developed by CIMMYT with collaboration of domestic research organizations.

S.no	Cultivar/variety name	Country (region)	Collaborating agencies/developers	Reported Zn increase vs local checks (approx.)	Reference
1.	BIO-MZN01	Colombia (LAC)	CIMMYT- HarvestPlus	~36% higher Zn than standard varieties	Xu et al., 2022
2.	ICTA B- 15	Guatemala (LAC)	CIMMYT- ICTA HarvestPlus	>60% higher Zn in tortilla products	Goredema-Matongera et al., 2021
3.	ICTA HB-18	Guatemala (LAC)	CIMMYT- ICTA HarvestPlus	>15% higher Zn than standard maize	Maqbool and Beshir (2018)

### Molecular breeding and quantitative genetic tools

The transition from conventional phenotype-based selection to molecular breeding represents a strategic pivot in biofortification. The driving force behind this shift is the need to bypass the phenotyping bottleneck the reliance on costly, low-throughput element quantification (ICP-OES), which constrains early-generation selection (Ndlovu et al., 2024). Molecular tools provide the resolution to dissect the polygenic architecture of Zn accumulation, allowing breeders to track quantitative trait loci (QTLs) associated with uptake, transport, and sequestration without destructive sampling (Basnet and Khanal, 2022; Baxter et al., 2013). Early bi-parental mapping efforts established the foundational landscape of Zn inheritance (Kaur et al., 2023). Consistent with Qin et al., 2012, CIMMYT-HarvestPlus trials identified reproducible loci across chromosomes 2, 3, 4, 5, 7, and 9. More recent studies have expanded this scope, Ndlovu et al., 2024 highlighted pleiotropic intervals influencing both protein and oil, while Xue et al., 2019 reported 21 loci specific to Zn-deficiency tolerance. Details of major QTLs for Zn accumulation in maize are presented in **Table 6**. However, a critical limitation of these primary QTL studies remains their broad confidence intervals (CIs) and high sensitivity to genotype-environment interactions. This instability often renders single-study QTLs unsuitable for marker-assisted selection (MAS), as a locus effective in one environment may vanish in another (Vasistha et al., 2024; Zheng et al., 2021).

**Table 6:** Details of major QTLs for Zn accumulation in maize are identified in different studies.

S.no	Mapping population	Population size	Marker type	Chromosome no	No of QTLs	LOD	References
1.	Inbred lines (GWAS)	244	SNP	7	202	–	Chen et al., 2024
2.	K22× BY815	209	SNP	1,2,3,5,6,7,9,10	21	3.0-5.7	Xu et al., 2022
3.	Ye478× Wu312 RIL	55	SSR	1,2,3,5,10	13	7.6-63.5	Xu et al., 2021
4.	RIL	–	SSR	2,3,4,5,6,7,8,9	22	3.01-31.94	Zhang et al., 2017
5.	F_2:3_ (178×P53)	218	SSR	2,5,10	4	3.01-5.58	Gu et al., 2015
6.	F_2:3_178/P53	218	SSR	2,5,10	4	3.01-5.58	Jin et al., 2013
7.	B73×Mo17	274	High-density	–	3	5-10	Baxter et al., 2013; Lungaho et al., 2011;
8.	Mo17×SDM	189	SSR	2,9,10	6	2.56-11.78	Qin et al., 2012
9.	B84×Os6-2	294	–	1	2	8	Simic et al., 2012
10.	DH8/DH40	–	SSR	2,3.4,6,7,10	4	2.15-5.23	Zhou et al., 2010

To mitigate this limitation, Meta-QTL (MQTL) analysis has emerged as a vital tool for consensus mapping. By aggregating independent results, MQTL refines peak positions (Sethi et al., 2023) and narrows CI widths (Gopinath et al., 2023), identifying MAS-friendly regions with high stability (Soriano and Alvaro, 2019). This utility was demonstrated by Sharma et al., 2023, who successfully prioritized intervals for fine-mapping grain Zn/Fe. By filtering out environment-specific noise, MQTL provides the statistical confidence required to validate candidate genes before investing in fine mapping (Tang et al., 2024). The integration of Meta-QTL (MQTL) analysis into breeding pipelines offers a robust mechanism for precision introgression. The operational value of this approach lies in its ability to tag consensus regions with diagnostic markers, thereby reducing the reliance on costly elemental phenotyping in early generations (Guo et al., 2013). Nevertheless, mapping discrete loci is only the first step. A key limitation of MQTL analysis is its dependence on publicly available QTL datasets, which differ in genetic background and developmental stage, hence requires validation (Hajibarat et al., 2024; Cabas-Luhmann et al., 2024). Furthermore, previous meta-analyses have largely focused on yield-related traits, leaving the genetic architecture of nutritional traits were remained underexplored. (Soriano et al., 2021). It is important to note that the majority of these QTLs were identified for Zn concentration in mature, dry kernels. Since baby corn is harvested at the immature silking stage, future research must validate, whether these same genomic regions also control Zn accumulation during the vegetative-to-reproductive transition, or if a distinct set of early-acting loci governs nutritional quality in vegetable maize. This key nutritional gap requires future validation.

By narrowing confidence intervals, MQTL-guided marker-assisted selection (MAS) ensures that major effect loci are prioritized for fixation. However, the exclusive reliance on MAS presents a strategic vulnerability due to QTL × environment interactions. As noted by Bernardo (2016), MAS has limited efficacy for quantitative traits like Zn concentration because it focuses solely on peak loci, while ignoring the genetic background. The physiological basis for this limitation is the polygenic nature of micronutrient accumulation, numerous small-effect loci, which are invisible to standard MAS, collectively account for a significant proportion of phenotypic variance (Joshi et al., 2023; Dong et al., 2015). Consequently, MAS often hits a genetic ceiling, failing to capture the minor alleles necessary for maximizing genetic gain (Truntzler et al., 2010).

To address the issue of missing heritability, genomic selection (GS) has emerged as a necessary complement. As described by Bernardo (2016), GS integrates genome-wide molecular marker data with phenotypic training populations to predict genomic estimated breeding values (GEBVs) for individual genotypes, thereby capturing the aggregate effects of numerous minor alleles. Unlike MAS, which targets specific loci, GS captures the cumulative contribution of the entire genome, even for lines with no phenotypic data (Crossa et al., 2017). This shift allows breeders to select for the background effect the crucial minor genes that stabilize Zn accumulation across environments. Therefore, the optimal path forward is not a choice between MAS and GS, but their integration. The results are in line with (Yan et al., 2023; Velu et al., 2016). Future breeding programs should adopt a tandem selection strategy, utilizing MAS/MABC to fix large-effect, validated MQTLs, while simultaneously applying GS models to select the highest-performing genetic backgrounds (Bernardo, 2016). A critical prerequisite for this strategy, however, remains the construction of robust, multi-environment training populations (TPs). Without a TP that accurately represents the target environment, the predictive accuracy of GS models for Zn will falter (Marques et al., 2021).

Genome-Wide Association Studies (GWAS) have become indispensable for overcoming the resolution limits of bi-parental mapping. The physiological basis for this advantage lies in the exploitation of historical recombination events accumulated over hundreds of generations in diverse panels. This process fractures linkage blocks, enabling the precise detection of small-effect loci (Liu and Yan, 2019; Yan et al., 2011). This perspective aligns with Chao et al., 2023, who demonstrated that GWAS is most powerful when employed to validate findings from bi-parental populations. For instance, Hindu et al., 2018 utilized large GWAS panels to identify SNPs for kernel Zn and Fe that were subsequently corroborated in mapping populations. This triangulation approach mitigates the risk of false positives caused by population structure a common pitfall in association mapping and provides validated entry points for candidate gene cloning. These results are consistent with findings reported by Zheng et al., 2018; Gupta et al., 2015; Yu et al., 2006.

Although the natural maize gene pool possesses substantial variability for Zn accumulation, it is often deficient in specific, high-value alleles required for bioavailability. In such cases, induced mutation breeding remains a practical route to generate novel variation (Collard and Mackill, 2008). However, the utility of this approach is often constrained by linkage drag and deleterious pleiotropy. Because chemical mutagenesis (EMS) is random, beneficial mutations in Zn transporters often come with heavy genetic loads that reduce yield or vigour (McMullen et al., 2009). Consequently, while mutation breeding has been effective historically, it is increasingly viewed as a brute force precursor to more precise methods. Modern pipelines now utilize TILLING primarily to identify targets for gene editing, rather than as a standalone breeding strategy (Shahzad et al., 2014).

Future breeding pipelines must move beyond simple MAS toward genomic selection (GS). While QTLs explain major variance, GS models capture the missing heritability of minor genes distributed across the genome. Consequently, the integration of genome-wide association studies (GWAS) for high-resolution allele mining, combined with GS for predicting breeding values, offers the most robust pathway for accelerating genetic gain in polygenic micronutrient traits. These results are further validated by Han et al., 2020; Liu and Yan, 2019; Wang et al., 2020; Zhang et al., 2018.

### Genetic engineering of Zn homeostasis

While conventional and molecular breeding can optimize existing genetic variation, they remain constrained by the fundamental biological boundaries of the maize gene pool. Genetic Engineering (GE) provides the necessary technological leap to transcend these limits, offering a targeted approach to manipulate traits, where natural variation is exhausted, non-existent, or chemically complex (Jha and Warkentin, 20200). Unlike recombination-based methods, GE enables the direct modulation of the rate-limiting steps of the Zn homeostasis network. This includes the overexpression of uptake transporters (*ZIP, NRAMP* families) to mine soil Zn (Von Wiren et al., 2000), the enhancement of chelator biosynthesis (Nicotianamine/Phytosiderophore pathways) for xylem mobility (Callahan et al., 2006), and the upregulation of loading regulators (*YSL, HMA*) for sequestration (Curie et al., 2001). The paradigm has been further shifted by the advent of genome-editing platforms such as Clustered regularly interspaced short palindromic repeats (CRISPR/Cas). These tools expand the breeder’s toolkit beyond transgenic overexpression, enabling footprint-free precision modifications (Teckchandani et al., 2024). These approaches offer high precision and can produce large effect changes, but they also bring regulatory, biosafety, and public-acceptance considerations (Mir et al., 2020). Moreover, engineered modifications require thorough functional validation to detect off-target or pleiotropic effects (Singh et al., 2016). The subsequent sections critically examine these strategies, categorized by their specific impact on uptake, transport, and sequestration.

#### Zn uptake at the soil-root interface

The acquisition of Zn from the rhizosphere is the primary rate-limiting step in biofortification, governed in maize by a sophisticated dual-uptake system (Tennstedt et al., 2009). Root influx is mediated through two distinct pathways depending on soil status: the direct uptake of ionic Zn (Zn^+2^) via high-affinity ZIP (Zrt-Irt-like Protein) transporters (Assuncao et al., 2010), and the strategy II pathway involving the secretion of Phyto siderophores (PS) specifically deoxymugineic acid (DMA), which chelate soil Zn for retrieval by YSL transporters (He et al., 2021). Under deficiency, this network exhibits high plasticity, upregulating key orthologs (e.g., ZmZIP3, ZmZIP4, and ZmZIP5) and biosynthetic genes to solubilize unavailable fractions (Swamy et al., 2021; Hindu et al., 2018). However, relying solely on this natural plasticity is insufficient for baby corn, while conventional breeding targets accumulation over a full 120-day season, baby corn faces a unique temporal challenge: maximizing uptake flux within a condensed vegetative window (approx. 45–50 days) (Khan et al., 2014). Furthermore, Curie et al., 2001 reported that efficacy of the PS-mediated pathway in the calcareous soils often used for maize production remains a validation gap. The engineering solution (CRISPR) this is, where genetic engineering offers a distinct strategic advantage. By utilizing CRISPR/Cas9 to target the promoter regions of these rate-limiting transporters (e.g., ZmZIP3 or ZmNAS), it is theoretically possible to enhance the uptake velocity of Zn during the rapid growth phase (Han et al., 2021; Haney et al., 2005). Unlike phytate-reduction strategies, which are redundant for baby corn metabolic engineering for hyper-accumulation represents the frontier. Consequently, characterizing and editing these transporter-chelator modules provides the most direct route to ensuring the immature ear reaches nutritional targets prior to harvest (Singh et al., 2017; Curie et al., 2001).

While ZmZIP genes are well-characterized in hydroponics, the extent to which Phyto siderophore-mediated uptake drives Zn acquisition in calcareous (high pH) soils where biofortification is most needed requires further functional validation. Although these mechanisms are well characterized in Arabidopsis and rice, functional validation of the corresponding maize orthologs (e.g., *ZmZIP1-ZmZIP8*, *ZmHMA*, *ZmMTP*, *ZmNAS*) remains limited (Vasconcelos et al., 2017; Tian et al., 2016).

#### Xylem-phloem transport and remobilization

The translocation of Zn from source to sink represents a complex physiological relay involving two distinct transport systems. Long-distance transport from roots to shoots occurs via the xylem, driven by transpiration pull, where Zn moves primarily as free ions (Zn^+2^) or bound to organic acids like citrate (Ma et al., 2024). However, the critical step for biofortification is the subsequent remobilization: the transfer of Zn from vegetative tissues (source) to the developing ear (sink) via the phloem (Kar et al., 2022). Unlike the xylem, phloem mobility is strictly chelate-dependent, relying on Nicotianamine (NA) and Mugineic Acid (MA) complexes to prevent precipitation in the high-pH phloem sap. The gatekeepers of this xylem-to-phloem transfer are the heavy metal ATPases (HMAs). Their pivotal role is well-established in model systems. Hou et al., 2022 demonstrated that the hma2/hma4 double mutant in Arabidopsis causes a severe root-shoot block, trapping Zn in the roots and reducing shoot accumulation by twofold. Conversely, overexpression strategies confirm this limitation, Wu et al., 2023 reported that overexpressing AtHMA4 enhances root efflux, doubling Zn concentrations in the leaves. In maize, this node-based transfer is the rate-limiting step. While HMAs load Zn into the xylem, the YSL (Yellow Stripe-Like) family is largely responsible for loading Zn-NA complexes into the phloem for grain filling (Song et al., 2024).

While the general maize ideotype emphasizes full-season remobilization, the unique physiology of baby corn harvested at the peak of vegetative-to-reproductive transition suggests that genetic engineering targets must be prioritized differently, focusing specifically on the upregulation of uptake transporters (e.g., ZmZIP3) during the rapid vegetative growth phase (Hossam-Abdelmonem et al., 2024).

#### Sequestration dynamics

In conventional maize physiology, kernel Zn accumulation is heavily dependent on the senescence-driven remobilization of reserves from vegetative tissues (R2-R4 stages). While estimates vary, substantial evidence indicates that in mature grain, a significant fraction of Zn is sourced from this internal recycling, particularly when soil uptake declines post-anthesis. This source-to-sink remobilization is a cornerstone of biofortification strategies for grain maize (Ibrahim et al., 2022).

However, baby corn production represents a fundamental physiological truncation of this process. Harvested at the onset of silking (stage R1) prior to the onset of vegetative senescence, baby corn is removed from the plant before the remobilization phase is triggered. Physiologically, this implies that the remobilization window is effectively closed (Fixen et al., 2010). Unlike mature maize, the immature ear must rely almost exclusively on concurrent root uptake and rapid xylem translocation. This physiological feasibility directly influences nutritional quality (Grennan, 2009). While mature maize requires milling and fermentation to reduce phytate barriers often resulting in restricted net Zn bioavailability despite high total concentrations baby corn is consumed whole with minimal processing. Because harvest occurs before phytate accumulation, Zn remains in a soluble, highly bioavailable ionic form (Fernandez and Hoeft, 2009). Furthermore, agronomic biofortification can further enhance this advantage by reducing crude fiber content.

Nevertheless, this unique harvest timing creates a significant gap in our agronomic understanding. Current partitioning models derived for mature grain are inherently invalid for baby corn. It is hypothesized that the sink strength of the immature cob is driven by immediate soil availability (flux) rather than stored reserves (Sawyer and Mallarino, 2007). Consequently, future research must focus on quantifying the Zn partitioning index specifically at the R1 stage, and determining if foliar applications at silking are more effective than basal dosing for this short-duration crop.

## Bioavailability determinants in baby corn

While the early harvest of baby corn effectively eliminates the phytic acid barrier, it introduces a distinct set of bioavailability constraints associated with the consumption of the entire ear. Unlike mature maize, where the fibrous hull is removed during processing, baby corn consumption involves the ingestion of the rachis (cob) and immature glumes (Pramitha et al., 2020). Consequently, the primary obstructive factor shifts from chemical chelation (phytate) to physical entrapment via structural carbohydrates. This hypothesis aligns with findings in other whole-vegetable matrices, suggesting that crude fibre specifically the hemicellulose and lignin components of the cob acts as a physical matrix (Thavaprakaash et al., 2005; Chand et al., 2016). Although less lignified than mature tissue, this fibre matrix can adsorb divalent cations and increase intestinal bulk, thereby reducing the residence time available for Zn absorption (Shanti et al., 2012). Interestingly, agronomic biofortification appears to mitigate this structural constraint. A recent study by Saikrishna et al., 2025 revealed that the combined application of Zn (soil + foliar) significantly reduced crude fibre content in the ear (lowering it from 5.8% in controls to 3.6% in treated plots), while simultaneously enhancing crude protein and sugar content. This implies a synergistic benefit, Zn application not only increases mineral density but also improves bioavailability by reducing the fibrous matrix that would otherwise sequester the nutrient. Results are in line with Akhtar et al., 2018; Bhoya et al., 2013.

A potential secondary limitation arises from the concentration of phenolic compounds and mineral competition. Immature maize tissues synthesize polyphenols for biotic defense, which possess hydroxyl groups capable of chelating Zn, albeit with lower affinity than phytate (Castro-Alba et al., 2019). Furthermore, high concentrations of competing divalent cations (Ca, Fe, and Cu) may inhibit Zn uptake through competitive transport at intestinal absorptive sites same findings are reported by Li et al., 2019; Xie et al., 2019; Kremper et al., 2015. Therefore, future nutritional profiling must extend beyond simple elemental analysis to characterize these fibre and polyphenol-Zn interactions to accurately predict the net bioavailability of Zn in biofortified baby corn for greater consumer acceptance.

## Economic rationale for Zn biofortification

The primary driver of global malnutrition is the unavailability and unaffordability of nutrient-dense diets, particularly in developing nations across Africa and Asia. It is estimated that over 3 billion people worldwide lack access to a healthy diet, creating a severe drag on national gross domestic product (GDP)s due to lost productivity (Sun et al., 2019; Xue et al., 2019). By 2030, diet-related health costs linked to non-communicable diseases are projected to exceed USD 1.3 trillion. In this context, biofortification represents a highly cost-effective public health intervention (Nakandalage et al., 2016). Unlike supplementation, biofortification is a one-time investment in breeding that delivers perpetual economic benefits, as improved varieties continue to produce nutrient-dense grain without recurring costs (Maqbool and Beshir, 2018). Economic analyses by global institutions, including the Copenhagen consensus, consistently rank biofortification among the highest-return development investments, with benefit-cost ratios ranging from 15:1 to 40:1, depending on the adoption rate and the severity of Zn deficiency in the target population (http://www.copenhagenconsensus.com/publication/second-copenhagen-consensus-biofortification-best-practice-meenakshi).

For smallholder farmers, adopting Zn-biofortified babycorn varieties generally requires no additional production cost, as these crops utilize the same agronomic practices as conventional maize varieties. Furthermore, cultivating Zn-efficient genotypes offers a distinct advantage, as they exhibit robust uptake and accumulation patterns even in Zn-deficient soils with good fertilization techniques (Adu et al., 2018). Wang et al., 2020 analyzed the cost-effectiveness of this approach using disability-adjusted life years (DALYs) to quantify health outcomes. They demonstrated that agronomic biofortification of wheat could reduce Zn deficiency-related health burdens by up to 56.6% in target regions. The study calculated that saving one DALY costs between US $226 and $594 when foliar Zn is applied alone. Crucially, when Zn application is combined with routine pesticide sprays, labour costs are shared, dropping the cost per DALY saved to between US $41 and $108. Key challenges, solutions, and future perspectives of Zn biofortification are presented in **Table 7**.

**Table 7:** Key challenges, underlying mechanisms, strategic solutions, and future perspectives for Zn biofortification.

S.no	Domain	Key challenge	Mechanism/underlying cause	Mitigation strategies & future prospective	References
1.	Agronomic	Edaphic & Management Variability	Soil heterogeneity (pH) and incorrect fertilizer sourcing lead to irregular Zn availability and fixation.	•**Precision Nutrient Management**: Adoption of chelated sources (Zn-EDTA) and nano-Zn fertilizers to enhance use efficiency. •Future focus on bio-priming with Zn-solubilizing microbes to unlock soil reserves.	Zaman et al., 2018
2.	Genetic (Breeding)	G × E Instability	Zn accumulation is a quantitative trait with variable heritability, expression varies significantly across different environments.	•**Stability Breeding**: Execution of multi-environment trials (MET) coupled with AMMI/GGE biplot analysis to identify stable donors. •Future integration of envirotyping to predict performance in untested locations.	Katral et al., 2024
		Yield-Nutrient Trade-off	A strong negative correlation exists between yield and mineral concentration (Dilution Effect). High-yielding hybrids often show reduced Zn density.	•**Genomic Selection (GS)**: Implementation of selection indices that assign economic weights to both yield and Zn density. •Use of CRISPR-Cas9 to break linkage drag between yield and nutrient loci.	Andersson et al., 2017
3.	Socio-Economic	The Invisible Trait Dilemma	Zn-maize lacks visual distinction. Farmers and consumers cannot verify Zn content, leading to low willingness-to-pay. Consumer acceptance is also limited because lack of knowledge on its advantages hinder its acceptance.	•**Nutri-Branding & Traceability**: Development of a distinct “Biofortified” certification logo to distinguish produce. •Integration into digital supply chains (Blockchain) to ensure traceability and build consumer trust despite the lack of visual difference.	Mir et al., 2020
4.	Policy & Regulatory	Regulatory hurdles & market lag	Strict regulatory frameworks for transgenic/genome-edited crops slow down commercialization. Lack of procurement incentives stalls farmer adoption.	•**Policy Harmonization**: Streamlining biosafety protocols for genome edited crops (SDN-1/SDN-2 categories). •Mandating procurement quotas in national food safety nets (e.g., India’s PDS) to guarantee a market sink for farmers.	Okwuonu et al., 2021

Governments must prioritize biofortification as a strategic investment in national health security rather than merely a nutritional subsidy. In the context of baby corn, this approach offers a dual socio-economic advantage, it functions as a high-value premium commodity with significant export potential that enhance farm livelihoods, while simultaneously strengthening local food systems through the supply of bioavailable Zinc (Mahapatra et al., 2018). Since biofortified crops are indistinguishable from conventional ones requiring no behavioural change from consumers they offer a sustainable, communal approach to eradicating hidden hunger, linking bottom-level grower economics with national well-being.

## Conclusion

Addressing the global burden of zinc malnutrition requires a systemic shift from calorie-centric production to nutrient-sensitive agriculture. In this context, biofortification offers a more practical and sustainable approach compared to supplementation, effectively ameliorating hidden hunger by delivering both yield and nutritional quality particularly in low-income regions. This review positions baby corn not merely as a high-value vegetable, but as a strategic, rapid-response vehicle for zinc biofortification. Its unique physiological advantage harvesting at the onset of silking effectively bypasses the phytic acid barrier that constrains zinc bioavailability in mature grain, thereby offering a superior bioavailable nutrient-to-calorie ratio for human consumption.

A synthesis of current evidence indicates that a dual-intervention strategy is requisite for success. Agronomically, the adoption of precision delivery systems specifically split-soil placement combined with stage-specific foliar sprays during the vegetative-to-reproductive transition (V10-R1) is critical to synchronize nutrient supply with the crop uptake flux. Simultaneously, breeding targets must evolve from simple concentration metrics to flux-based ideotypes, utilizing genomic tools to upregulate transporter activity specifically during the condensed vegetative window. Ultimately, the integration of these conventional strategies with emerging technologies including microbial bio-inoculants, nano-fertilizers, and CRISPR/Cas9 editing offers the most sustainable pathway to alleviating zinc malnutrition.

However, future success now hinges on harmonizing these scientific advances with robust policy support, creating market-linked supply chains that explicitly value nutritional density alongside agronomic yield. Furthermore, it requires cross-disciplinary convergence bridging plant physiology, human nutrition, and agricultural economics to maximize public health outcomes and economic prosperity in alignment with the UN Sustainable Development Goals (SDGs).
